# High-resolution forest mapping for behavioural studies in the Nature Reserve ‘Les Nouragues’, French Guiana

**DOI:** 10.1080/17445647.2014.972995

**Published:** 2014-10-28

**Authors:** Max Ringler, Rosanna Mangione, Andrius Pašukonis, Gerhard Rainer, Kristin Gyimesi, Julia Felling, Hannes Kronaus, Maxime Réjou-Méchain, Jérôme Chave, Karl Reiter, Eva Ringler

**Affiliations:** a University of Vienna, Department of Integrative Zoology, Vienna, Austria; b Haus des Meeres – Aqua Terra Zoo, Vienna, Austria; c University of Vienna, Department of Cognitive Biology, Vienna, Austria; e Grammar School GRG11, Vienna, Austria; f Université Paul Sabatier, Laboratoire Evolution et Diversité Biologique, Toulouse, France; g University of Vienna, Department of Botany and Biodiversity Research, Vienna, Austria

**Keywords:** Compass mapping, digital map, tree mapping, forest inventory, Neotropical rainforest

## Abstract

For animals with spatially complex behaviours at relatively small scales, the resolution of a global positioning system (GPS) receiver location is often below the resolution needed to correctly map animals’ spatial behaviour. Natural conditions such as canopy cover, canyons or clouds can further degrade GPS receiver reception. Here we present a detailed, high-resolution map of a 4.6 ha Neotropical river island and a 8.3 ha mainland plot with the location of every tree >5 cm DBH and all structures on the forest floor, which are relevant to our study species, the territorial frog *Allobates femoralis* (Dendrobatidae). The map was derived using distance- and compass-based survey techniques, rooted on dGPS reference points, and incorporates altitudinal information based on a LiDAR survey of the area.

## 1. Introduction

**Figure 1:**
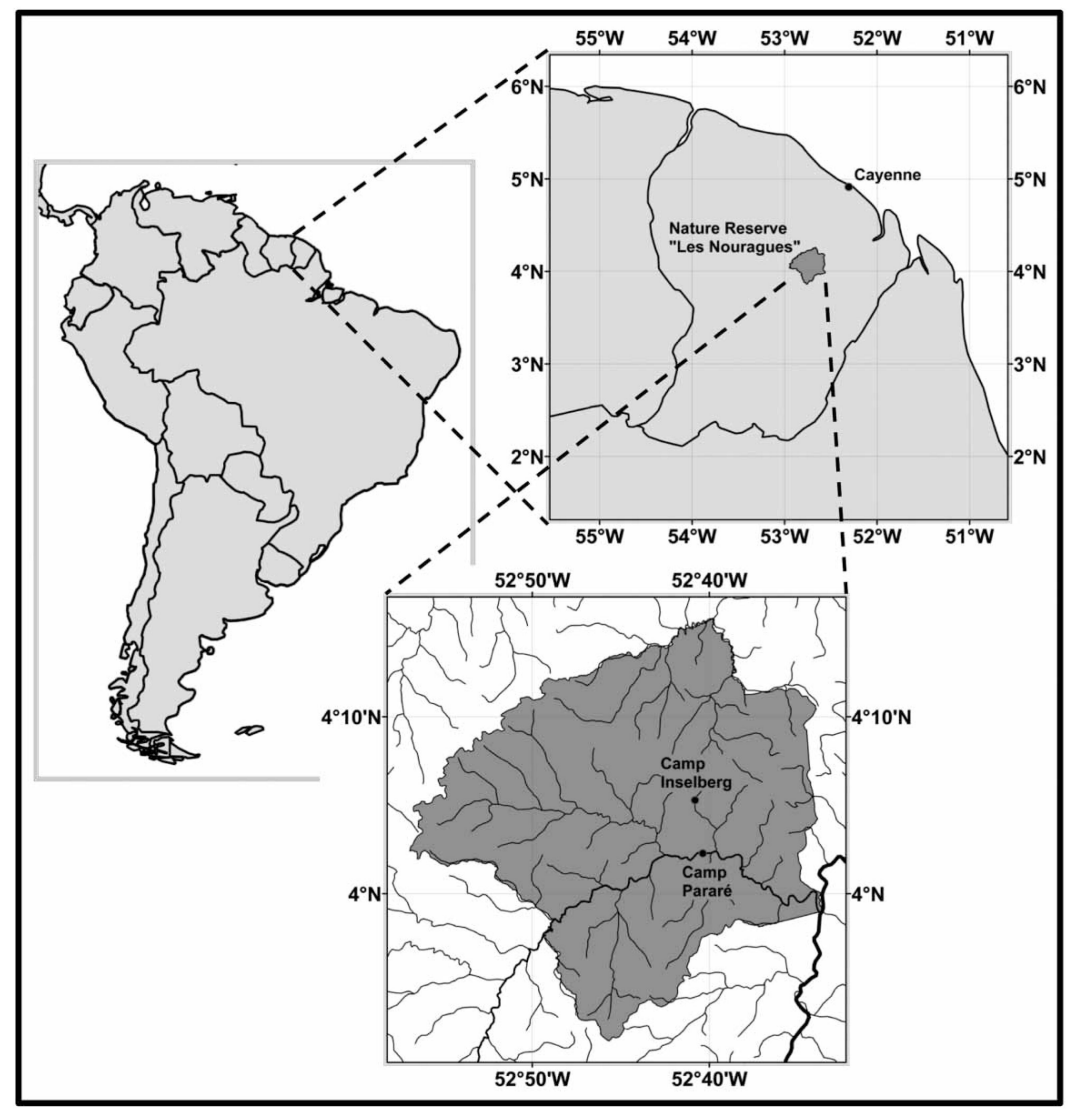
Location of the research camps ‘Inselberg’ and ‘Saut Pararé’ in the ‘Les Nouragues’ nature reserve; lines in the insert of the nature reserve represent the drainage system, with the rivers ‘Approuague’ (major) and ‘Arataye’ (minor) in bold. Outline of the nature reserve and drainage system data were provided by the French National Forest Agency (Office National des Forêts, ONF), the world borders dataset was obtained from www.thematicmapping.org under the creative commons attribution-share alike license 3.0.

**Figure 2:**
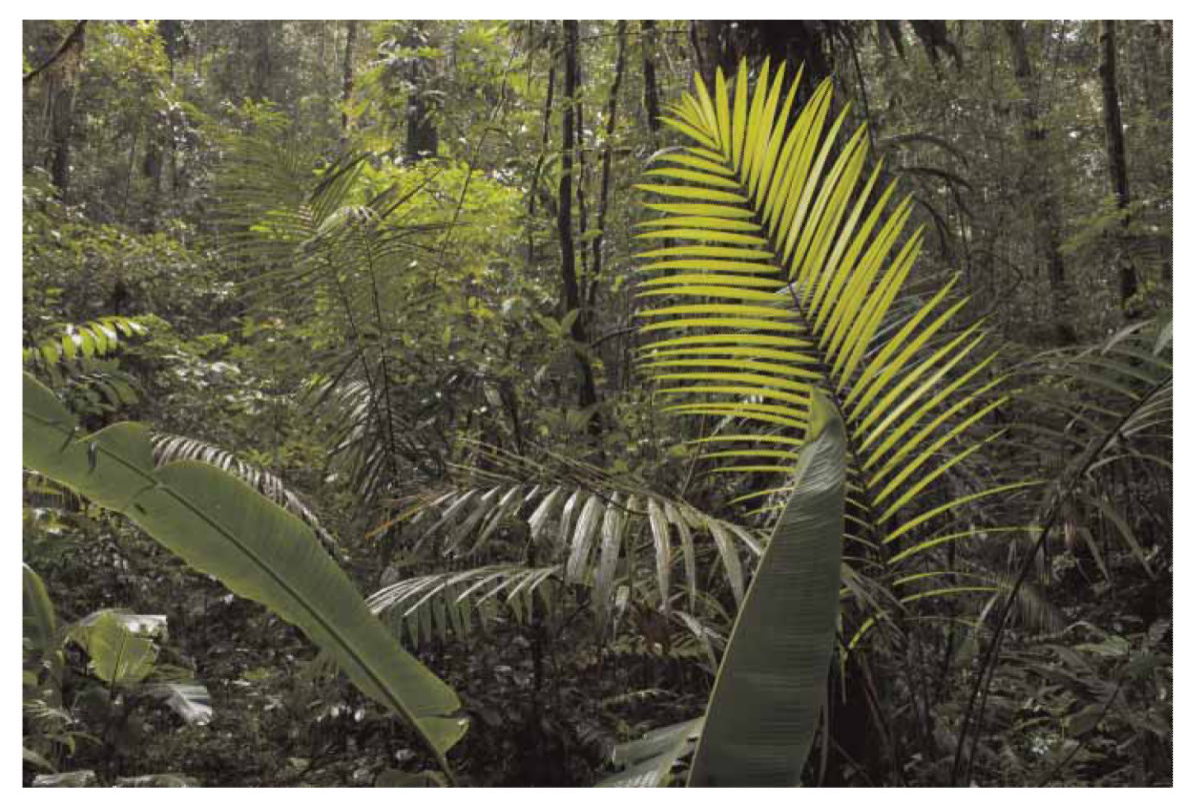
Typical forest understory at the Pararé site with *Astrocaryum paramaca* palms.

The Nouragues nature reserve (http://www.nouragues.fr) in the French overseas department French Guiana is a protected area under national law since 1995. The reserve hosts the Nouragues Ecological Research Station (http://www.nouragues.cnrs.fr/), run by the CNRS (Centre National de la Recherche Scientifique), with the two camps: ‘Inselberg’ (4°05′N, 52°41′W) and ‘Saut Pararé’ (4°02′N, 52°41′W). The Inselberg site was established in 1986, prior to the creation of the reserve, and the Pararé site was established in 1997. Both camps are only accessible by helicopter or boat (Figure 1). The predominant vegetation type at the ‘Pararé’ site is primary lowland rainforest with a complex relief of small hills and ridges (27–80 m a.s.l.) that are partitioned by several small creeks. The most prominent plant species in the forest understory is the spiny palm *Astrocaryum paramaca* with densities up to 300 individuals/ha (Figure 2). A detailed characterisation of the study area is given in Bongers, Charles-Dominique, Forget, and Théry (2001).

**Figure 3:**
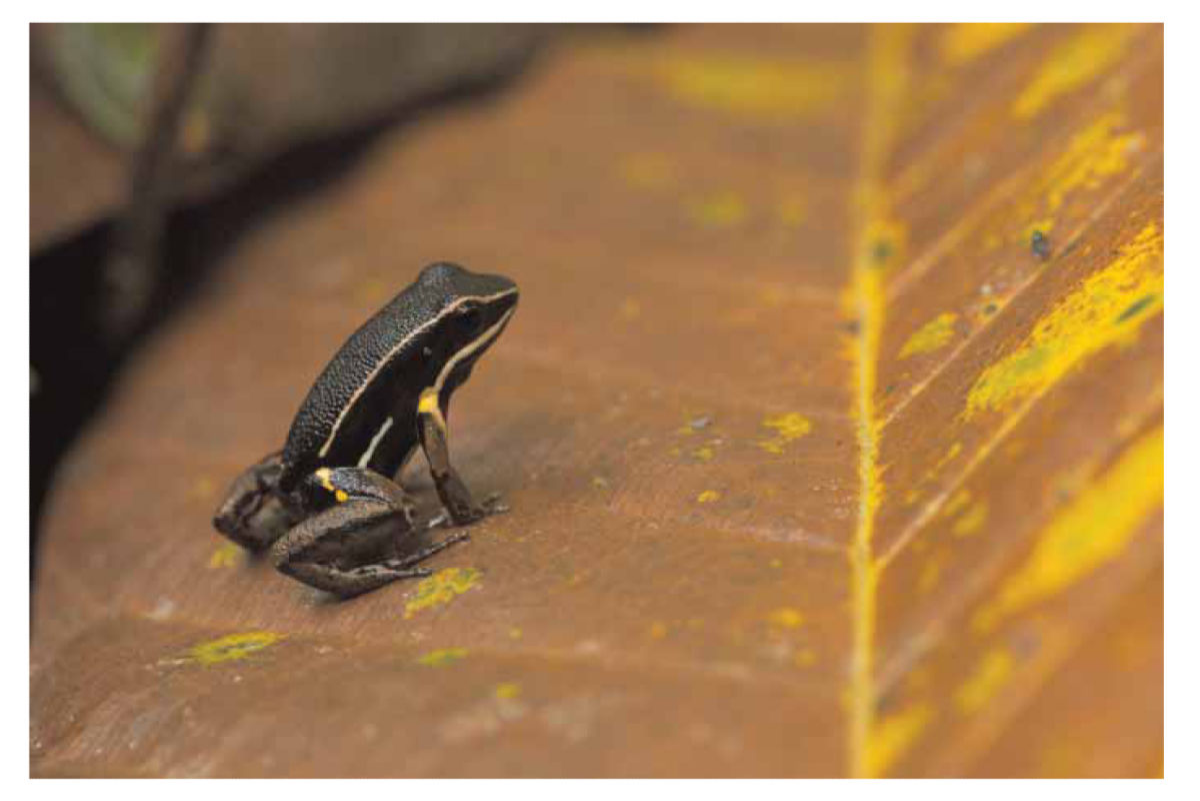
The Brilliant-Thighed Poison Frog *Allobates femoralis* (Dendrobatidae).

In 2008 we initiated an ongoing, long-term population study on the territorial, ground living frog *Allobates femoralis* (Figure 3) at the ‘Pararé’ site in a mainland plot of approximately 8.3 ha in the forest approximately 300 m from the field camp. The plot is bordered by the river Arataye to the south and two small creeks in the west and east. Towards the north, the plot extends for approximately 450 m along an ascending ridge. In 2012 we extended this work to a 4.6 ha river island, which is located approximately 45 m across from the field station in the river Arataye, to establish a controlled and closed population of *A. femoralis* for experimental work. Our population monitoring requires the detailed mapping of territories and movement of individual frogs, and given the poor global positioning system (GPS) resolution under the forest canopy (Ordóñez Galán, Rodríguez Pérez, García Cortés, & Bernardo Sánchez, 2013; Ordóñez Galán, Rodríguez-Pérez, Martínez Torres, & García Nieto, 2011; Wing & Frank, 2011) we decided against its use. Our preliminary trials with a survey-grade differential GPS device showed that the mapping resolution was ~10–20 m (data averaging over 30 sec). As *A. femoralis* males typically have territories with a diameter of 13.9 m when approximated as a circle (Ringler, Ringler, Magaña Mendoza, & Hödl, 2011), the GPS would not have allowed us to map the spatial behaviour of our study species inside its territories with the desired resolution of 50 cm. Therefore, we opted for a conventional, distance- and compass-based, forest inventory in the survey areas. It included the mapping of forest floor structures in order to obtain a highly detailed background map (see Main Map) that would allow us to directly identify and register frog positions on the map without further measurements needed. Beyond the original purpose of this project, we believe that such high-resolution maps can be of considerable value in forest ecology and animal behaviour at this site.

## 2. Methods and results

To provide the control point framework for our map we used survey-grade dGPS devices (Ashtech MobileMapper 10) with post-processing differential GPS correction (reference stations: Kourou, French Guiana; Zanderij, Suriname) to obtain global coordinates of two local points of origin for the mainland and island plots, respectively. At each point of origin, GPS data were recorded for five hours and for final positioning the average of the dGPS corrected locations was taken. The altitude a.s.l. at these points was obtained from a 1 m raster digital elevation model (DEM) that was derived from an airborne laser (LiDAR) scan of the area conducted in 2012 for a study on forest structure (for details see Kennel, Tramon, Barbier, & Vincent, 2013; Réjou-Méchain, Tymen, Blanc, Fauset, Feldpausch, Monteagudo, Phillips, Richard, & Chave, 2014; Vincent *et al.*, 2012). From these control points, we established a network of reference points, which were oriented by measuring the distance, compass direction and inclination from a known reference point to the next, newly established reference point (Bitterli, 1995; Dasher, 1994). Distance was measured with laser rangefinders (Bosch DLE 50) and recorded with an accuracy of 1 mm. Compass direction and inclination were measured with a combined precision compass and inclinometer (Suunto Tandem™ 360PC/360R DG, magnetic inclination zone 2) with an accuracy up to 0.5°. Both devices were mounted on a lightweight carbon tripod (Velbon Sherpa). We made sure the tripod did not contain any magnetic parts that could influence the compass. The instruments on the tripod were set up at the measuring stations using a brass plumb to adjust it to a defined height above ground, which varied with the body height of different instrument operators. The instruments were aimed at a height-adjustable cross-haired target disk which was hand held at the location of the new reference point. Data collection was conducted with MobileMappers and PocketPCs (HP iPAQ hx4700 & rx1950) running the mobile GIS software Esri ArcPad. Measurements were entered directly into ArcPad, using the ‘1-point offset’ function which calculates the 3-D position of an unknown target, based on a known reference point and distance, direction, and inclination measurements. Reference points were marked with labelled PVC tubes, which were 30/50 cm high and had a diameter of 16 mm. They were set arbitrarily in the area with typical intervals of 10–15 m, based on inter-point visibility, which was influenced by vegetation and relief. While establishing the network of reference points, we regularly crosschecked and corrected previous reference points from other adjacent reference points.

**Figure 4:**
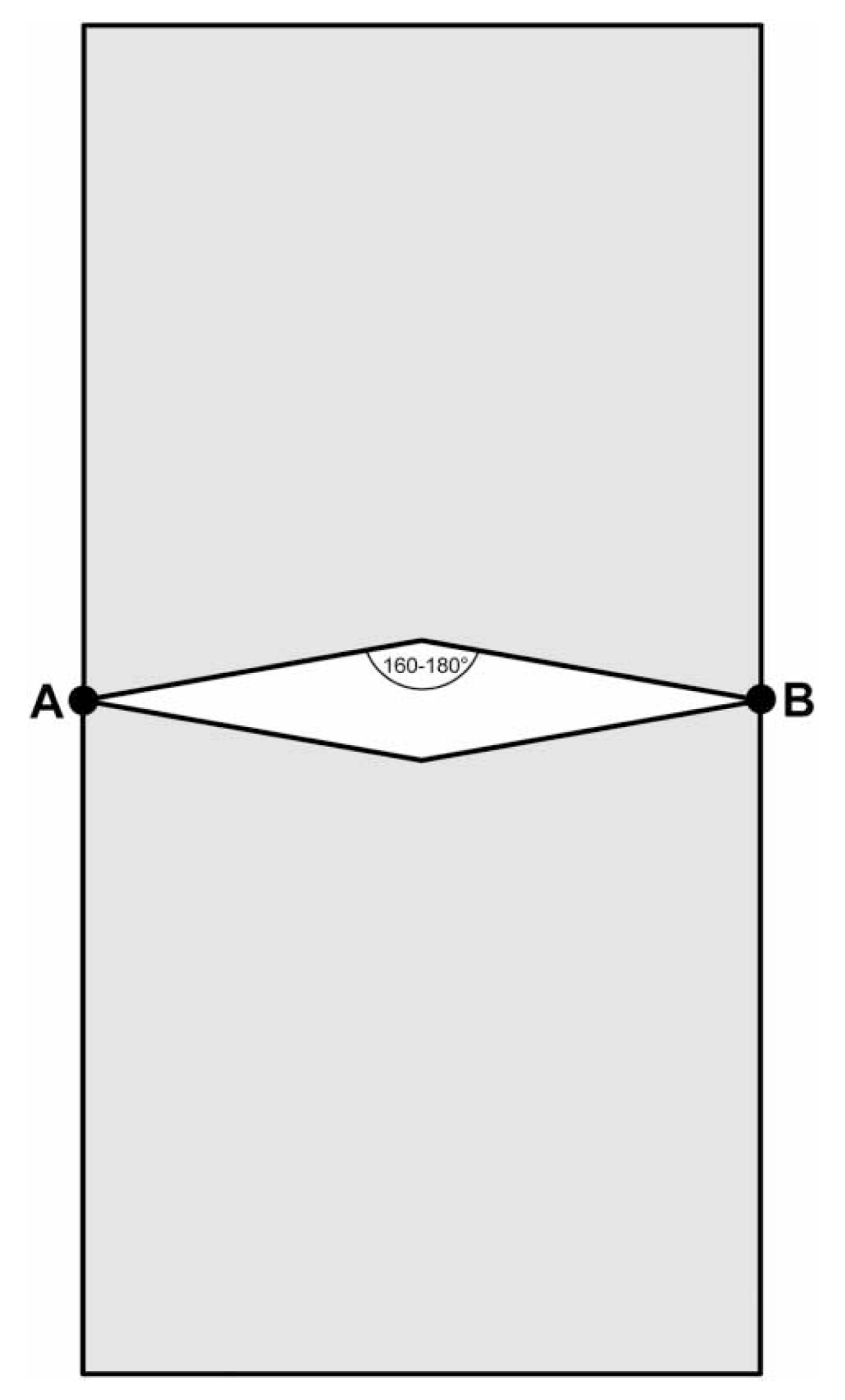
Approximate area (grey) where trees were measured from two reference points A and B.

Once the network of mapped and labelled reference points was established, we mapped trees and ground structures within direct line-of-sight from the reference points. Tree mapping required only 2-D locations as tree locations were later projected on the DEM to obtain 3-D orientation. Therefore we used the ‘2-point offset’ function in ArcPad to calculate the positions of tree-trunk centres, based on two known reference points and the intersection of two compass bearings from these points. We used two precision compasses which were mounted on de-magnetised tripods and set up at defined heights at two reference points with a direct line of sight to a given tree. From any two reference points we mapped a section of trees, which were approximately in the quadrat on both sides of these reference points, leaving out the area directly between the reference points, where the compass bearings would have intersected at angles between 160° and 180°. This minimised errors resulting from intersections that would have been obtuse (Figure 4). We mapped all trees with a diameter at breast height (DBH) >5 cm and estimated their DBH or the diameter above basal irregularities such as buttresses, and the diameter at ground level to the next 5 cm. To avoid repeated mapping of trees, we marked mapped trees with a thin white cotton thread around the trunk.

During tree mapping and later during the frog population study, we mapped all ground structures that were relevant to our study animals. This included entire fallen trees, large branches (>cm10 cm diameter), piles of woody debris like fallen treetops and liana thickets, roots running overground, aerial logs (broken trees, suspended by standing vegetation), and large palm leaves and spathae. We used the reference points and mapped trees as spatial reference to directly draw ground structures in the digital map in ArcPad; for large structures (e.g. fallen trees) we used offsetting in ArcPad to identify the extreme points of this object and then refined the outline by directly drawing on the digital map.

Our forest inventory and ground structure mapping resulted in a highly detailed background map (see Main Map) which was subsequently used to register the locations of individuals of our study species by directly drawing on the digital map. The map has thus been used in *A. femoralis* studies on the species’ mating system and population genetics (Ringler, Ringler, Jehle, & Hödl, 2012; Ursprung, Ringler, Jehle, & Hödl, 2011a), parental care (Ringler, Pašukonis, Hödl, & Ringler, 2013), territorial behaviour (Ringler et al., 2011), orientation and homing behaviour (Pašukonis *et al.*, 2013; Pašukonis, Loretto, Landler, Ringler, & Hödl, 2014), and longevity and effects of toe-clipping (Ursprung, Ringler, Jehle, & Hödl, 2011b). A study on turtles and tortoises was also conducted in the mainland plot and used parts of the map for locating Twist-Necked Turtles *Platemys platycephala* (Böhm, 2013a) and Yellow-Footed Tortoises *Chelonoidis denticulata* (Böhm, 2013b).

## 3. Conclusions

Our mapping approach proved to be very efficient in allowing us to obtain a highly detailed background map at a speed of approximately 1-ha per 7 days for a 3–4 person team and outperformed dGPS-based approaches. The use of compasses and consumer-grade tripods was preferable over theodolite mapping and surveyor-grade equipment given the moist environment, causing equipment damage or failure, and as the very short line of sight inside the forest resulted in the frequent relocation and re-setup of measuring stations, a task which would have been especially time consuming with a theodolite (cf. Condit, 1998; Vincent et al., 2012). Hence our method was a tradeoff decision between equipment costs and risks of equipment damage, availability of work power and time, relief and vegetation structure, and the precision and detail desired for the final background map. A retrospective manual control of the 3-D locations of our reference points against the LiDAR-DEM showed good accuracy for our mapping, which was far beyond the resolution obtainable by GPS inside the forest, even with differential post-processing.

Considering that this study was undertaken in a high-biodiversity area, with a heterogeneous terrain and complex understory and forest floor, the procedures described should be reproducible in other ecosystems and regions. Hence we suggest the use of our mapping technique for all studies on spatial behaviour that require a level of detail that cannot be obtained with GPS, where GPS resolution is coarser than the investigated movements of the study animals.

### Software

Field data were collected using Esri ArcPad with the GPS raw-data recording plug-in Ashtech GPSdifferential for ArcPad 7/8/10. Differential GPS post-processing was performed using Ashtech MobileMapper Office 2.1.5. GIS data handling and map drawing was conducted using Esri ArcGIS 9.3/10.0.

### Data

All data displayed in the maps are available for free use at Dryad: doi:10.5061/dryad.175cv The shapefiles will be updated with ongoing fieldwork at Camp Pararé and current versions can be requested from the corresponding author.
